# Multicentre Evaluation of Loop-Mediated Isothermal Amplification (LAMP) for Environmental Monitoring of Nosocomial Pathogens in Italian Hospitals

**DOI:** 10.3390/antibiotics15101012

**Published:** 2026-10-12

**Authors:** Biagio Santella, Antonio Donato, Vittoria Satriani, Laura Grumiro, Stefano Stracquadanio, Emanuela Santoro, Luigi Fortino, Claudia Colosimo, Alice Nicolosi, Maddalena Calvo, Graziella Manciagli, Zirone Nunziata, Agata Silvana Trifilò, Maria Giuseppina Pecora, Giuseppe Arcoleo, Monica Cricca, Stefania Stefani, Vittorio Sambri, Giovanni Boccia

**Affiliations:** 1 Department of Chemistry and Biology, University of Salerno, 84084 Fisciano, Italy; 2 Department of Medicine, Surgery and Dentistry “Scuola Medica Salernitana”, University of Salerno, 84081 Baronissi, Italy; adonato@unisa.it (A.D.); vsatriani@unisa.it (V.S.); esantoro@unisa.it (E.S.); lfortino@unisa.it (L.F.); 3 Department of Medical and Surgical Sciences (DIMEC), University of Bologna, 40138 Bologna, Italy; laura.grumiro@auslromagna.it (L.G.); claudia.colosimo2@unibo.it (C.C.); vittorio.sambri@unibo.it (V.S.); 4 Laboratory of the Greater Romagna Hub, 47522 Pievesestina, Italy; 5 Department of Medicine and Surgery, “Kore” University of Enna, 94100 Enna, Italy; stefano.stracquadanio@unikore.it; 6 Department of Biomedical and Biotechnological Sciences, University of Catania, 95123 Catania, Italystefania.stefani@unict.it (S.S.); 7 University Hospital Policlinico-San Marco, University of Catania, 95123 Catania, Italy; 8 National-Level and Highly Specialized Hospital “GARIBALDI” (ARNAS), 95123 Catania, Italy; 9 Enbiotech s.r.l., 90129 Palermo, Italy; 10 Department of Health Hygiene and Evaluative Medicine, A.O.U. San Giovanni di Dio e Ruggi d’Aragona, 84131 Salerno, Italy

**Keywords:** hospital environmental monitoring, healthcare-associated infections, LAMP, nosocomial pathogens, *Staphylococcus aureus*, *Pseudomonas aeruginosa*, *Klebsiella pneumoniae*, *Acinetobacter baumannii*

## Abstract

Background: Environmental contamination of hospital surfaces, especially in high-risk clinical areas, partly sustains healthcare-associated infections. The detection of microbiological contamination in hospital settings using culture-based methods remains fundamental, even though these methods are not rapid, show moderate sensitivity in areas with low microbial loads, and are unsuitable for some microbial species. This multicentre study evaluated the performance of loop-mediated isothermal amplification (LAMP) for the rapid detection of major nosocomial pathogens on hospital surfaces. Methods: A multicentre observational method–comparison study was conducted in three Italian hospital centres. Overall, 298 environmental surface samples were collected from clinical areas included in local infection-prevention monitoring plans. For each sampling point, LAMP swab sampling was paired with an adjacent contact plate culture. LAMP assays targeted *Staphylococcus aureus*, *Acinetobacter baumannii*, *Klebsiella pneumoniae*, and *Pseudomonas aeruginosa*. Manual and automated nucleic acid extraction workflows were analysed separately. Diagnostic performance, agreement metrics, and discordant paired results were assessed using a contact plate culture as the microbiological comparator. Results: No LAMP-negative/RODAC-positive pairs were observed. Positive percent agreement (PPA) relative to contact plate culture was therefore 100% in the subgroups evaluated. Relative specificity ranged from 90.9% to 98.6%, while overall accuracy ranged from 93.8% to 98.6%. Discordant results were exclusively LAMP-positive/contact plate-negative and were more frequent for *S. aureus* and *P. aeruginosa*. Selective culture verification supported several LAMP-positive findings not recovered by routine contact plates. Conclusions: LAMP showed high negative agreement, high specificity, and rapid target-specific detection of selected nosocomial pathogens on hospital surfaces. It may complement conventional cultures in environmental surveillance, particularly when timely feedback is needed to guide infection-prevention and sanitation-monitoring strategies.

## 1. Introduction

Healthcare-associated infections (HAIs) remain a major challenge for modern health systems, contributing significantly to morbidity, mortality, and resource consumption worldwide [1,2]. The World Health Organization estimates that around 7% of hospitalised individuals in affluent nations and as many as 15% in low- and middle-income nations are expected to acquire at least one hospital-associated infection during hospitalization. Microbiologically contaminated surfaces serve as reservoirs for bacteria responsible for HAIs, particularly in high-risk areas such as intensive care units, operating theatres, hematology and oncology wards, and infectious diseases units [3,4].

High-touch surfaces in the near-patient environment, including bed rails, infusion pumps, monitors, and worktops, can harbor pathogenic bacteria capable of surviving for prolonged periods, especially under suboptimal cleaning and disinfection practices. This contamination acts as a reservoir from which microorganisms may be transferred via healthcare workers’ hands or medical devices [5,6].

Among the most relevant nosocomial pathogens capable of resisting environmental decontamination are *Staphylococcus aureus*, *Acinetobacter baumannii*, *Klebsiella pneumoniae*, and *Pseudomonas aeruginosa*. These species are frequently multidrug-resistant, capable of forming biofilms, and particularly adept at persisting on dry surfaces [7]. Environmental microbiological monitoring, therefore, constitutes a fundamental pillar of HAI prevention and control, necessary both to evaluate decontamination and sanitization procedures and to identify resistant species that may require specific, appropriate interventions [8,9].

Currently, the culture method is considered the gold standard for detecting microorganisms on surfaces, such as contact plates or swabs plated on non-selective media, followed by incubation and identification of colonies by biochemical methods or MALDI-TOF mass spectrometry [10,11]. This method, while thorough, is unfortunately time-consuming, requiring 48 to 72 h for the observation and identification of bacterial species on the plate. Moreover, it involves a complex microbiological process that demands significant effort. As a result, it is not suitable for non-culturable microorganisms or those present in very low loads. Recent scoping work on cleaning–monitoring strategies has highlighted both the central role of microbiological methods and the need for faster, more sensitive tools integrated within multimodal surveillance frameworks [12,13].

Loop-mediated isothermal amplification (LAMP) is a nucleic acid amplification technique that operates at a constant temperature (typically 60–65 °C), using a set of four to six primers recognizing multiple regions of the target gene and generating large amounts of DNA within a short time [14,15]. LAMP offers several advantages for environmental monitoring: high analytical sensitivity and specificity, rapid turnaround (often ≤1 h), relatively simple workflows, and the possibility of visual or instrument-based readout [16].

LAMP assays have been developed for a wide range of microorganisms and applied in water safety, food safety, clinical diagnostics, and, more recently, environmental surveillance of SARS-CoV-2 and other pathogens [17].

A recent experimental study optimized LAMP for nosocomial pathogens on artificially contaminated surfaces, showing high sensitivity and specificity versus contact plate culture after short pre-incubation periods [18]. However, evidence from real hospital environments remains limited, especially for multicentre studies using routine high-touch surfaces in wards with differing risk levels and sanitation protocols.

The present multicentre study was designed to address this gap by evaluating the performance and practical feasibility of LAMP-based assays for the detection of *S. aureus*, *A. baumannii*, *K. pneumoniae*, and *P. aeruginosa* on hospital surfaces. Environmental samples were collected from three Italian hospitals across different regions and risk settings and analyzed in parallel by LAMP and RODAC contact plate culture methods, with a selective plating protocol used to verify LAMP-positive findings. The aim was to characterize the sensitivity, specificity, and turnaround time of LAMP and to explore its added value for environmental surveillance within infection prevention programs.

## 2. Results

### 2.1. Environmental Sampling

During the study period, 298 environmental surface samples were collected from three Italian regions: 132 samples in Campania, 93 in Emilia-Romagna, and 73 in Sicily. Sampling was performed on hospital surfaces selected according to local environmental monitoring plans and infection risk profiles, according to local monitoring plans. For each sampling point, LAMP swab sampling was paired with an adjacent RODAC/contact plate culture, allowing direct comparison between the molecular and culture-based approaches under real-world hospital conditions.

The final analytic performance dataset included 153 valid paired comparisons per target pathogen for manual extraction and 145 valid paired comparisons per target pathogen for automated extraction. Results were analysed separately for *S. aureus*, *A. baumannii*, *K. pneumoniae*, and *P. aeruginosa*, and were summarized according to the directly observed paired categories LAMP+/culture+, LAMP+/culture−, LAMP−/culture+, and LAMP−/culture−.

### 2.2. Analytical Performance of LAMP

Overall, no LAMP-negative/RODAC-positive results were observed for any target pathogen or extraction method. Relative sensitivity percent agreement was therefore 100% in all evaluable pathogen extraction subgroups. Negative predictive value was also 100% across all comparisons, indicating that LAMP did not miss any culture-positive sample in the available dataset. Because several targets had a low number of comparator-positive samples, particularly *A. baumannii* and *K. pneumoniae*, sensitivity estimates should be interpreted together with their confidence intervals and with caution.

Relative specificity percent agreement remained high across the study, ranging from 90.9% to 98.6%. Overall accuracy ranged from 93.8% to 98.6%, while balanced accuracy remained consistently high, ranging from 95.5% to 99.3%. The complete diagnostic performance and agreement metrics according to pathogen and extraction workflow are reported in Table 1.

### 2.3. Performance According to Extraction Workflow

With manual extraction, no LAMP-negative/RODAC-positive pairs were observed; however, several pathogen-specific PPA estimates were based on very small positive denominators and should be interpreted cautiously. Specificity ranged from 90.9% for *S. aureus* to 98.0% for *A. baumannii*, and accuracy ranged from 94.1% to 98.0%. The agreement was good to excellent, with Cohen’s κ values ranging from 0.760 to 0.876 and PABAK values from 0.882 to 0.961. The strongest agreement was observed for *S. aureus* (κ = 0.876) and *P. aeruginosa* (κ = 0.825), the two targets with the highest number of comparator-positive samples.

With automated extraction, relative sensitivity percent agreement remained 100% in all evaluable target subgroups, and specificity ranged from 93.5% for *S. aureus* to 98.6% for *A. baumannii*. Accuracy ranged from 93.8% to 98.6%, and PABAK remained high across targets (0.876–0.972), supporting substantial overall agreement after adjustment for prevalence imbalance. However, Cohen’s κ was lower for low-prevalence targets, particularly *K. pneumoniae* (κ = 0.326) and *P. aeruginosa* (κ = 0.393), reflecting the limited number of comparator-positive samples rather than a high number of false-negative LAMP results.

### 2.4. LAMP-Positive/Contact Plate-Negative Discordant Results

Discordant results were represented exclusively by LAMP-positive/RODAC-negative samples, whereas no LAMP-negative/RODAC-positive results were observed. This pattern indicates that LAMP did not fail to detect any culture-positive sample but yielded additional positive signals compared with routine contact plate culture. These additional LAMP-positive findings resulted in higher positivity rates for LAMP than for contact plates across all targets and extraction workflows.

For manual extraction, the absolute increase in positivity rate for LAMP compared with contact plates was +5.9% for *S. aureus*, +2.0% for *A. baumannii*, +2.6% for *K. pneumoniae*, and +4.6 for *P. aeruginosa*. The McNemar exact test showed significant asymmetric discordance for *S. aureus* (*p* = 0.004) and *P. aeruginosa* (*p* = 0.016), driven by LAMP-positive/contact plate culture-negative results. For automated extraction, the corresponding increase in the positivity rate was +6.2% for *S. aureus*, +1.4% for *A. baumannii*, +2.8% for *K. pneumoniae*, and +2.1% for *P. aeruginosa*. Significant asymmetric discordance was observed only for *S. aureus* (*p* = 0.004), whereas the remaining automated extraction comparisons were not statistically significant.

These findings should not be interpreted as evidence of diagnostic equivalence between methods. Rather, they show that discordance was directional and mostly reflected additional LAMP-positive findings. In the environmental monitoring of surfaces, results may stem from low-level contamination, diminished cultivation capacity, uneven microbial distribution on surfaces, or the presence of stressed cells or residual sanitizer.

### 2.5. Target Distribution Across Centres

The distribution of target pathogens varied across centres and extraction workflows. In the manual extraction dataset, valid comparisons were available for 47 samples from Cesena, 32 from Catania, and 74 from Salerno for each target. In the automated extraction dataset, valid comparisons were available for 46, 41, and 58 samples, respectively. *S. aureus* was the most frequently detected target overall, particularly in the manual extraction dataset, followed by *P. aeruginosa*, *A. baumannii,* and *K. pneumoniae* were detected less frequently and were characterized by a low number of comparator-positive samples (Supplementary Table S1).

Because sampling was performed according to centre-specific monitoring needs and included different wards, surfaces, and sanitation conditions, centre-level results were interpreted descriptively. Therefore, differences in target distribution across centres should be regarded as local detection patterns rather than direct prevalence comparisons between hospitals.

### 2.6. Selective Verification of LAMP-Positive Findings

Selective plating of enrichment broths corresponding to LAMP-positive samples was used as a microbiological verification step, particularly for LAMP-positive/RODAC-negative results. This verification approach was useful when routine non-selective contact plates showed no target pathogen recovery or when target colonies could have been masked by non-target environmental flora. Available verification results supported several LAMP-positive findings and reinforced the interpretation of LAMP-positive/contact plate culture-negative samples as biologically relevant discordant results rather than automatic analytical false positives (Supplementary Table S2).

Overall, the results indicate that LAMP provides rapid, pathogen-specific detection with high negative agreement against contact plate culture and the ability to identify additional target-positive signals not recovered by routine surface culture. These findings support its role as a complementary molecular tool for hospital environmental surveillance, particularly in settings where rapid feedback is needed for infection prevention and sanitation monitoring.

## 3. Discussion

### 3.1. Principal Findings

This multicentre real-world study evaluated the performance of a LAMP-based workflow for the rapid detection of four clinically relevant nosocomial pathogens on hospital environmental surfaces. The main finding was the absence of LAMP-negative/contact plate-positive results across all target pathogens and extraction workflows. Accordingly, relative sensitivity percent agreement was 100% in all evaluable comparisons. This result indicates that, within the limits of the culture-based comparator used in this study, LAMP did not miss any culture-positive sample. At the same time, relative specificity percent agreement remained high, ranging from 90.9% to 98.6%, and negative predictive value was 100% for all targets and extraction methods. These findings support the potential role of LAMP as a rapid screening tool in environmental microbiological surveillance, particularly when the priority is the early exclusion of contamination by selected high-risk pathogens [16,19].

A second key finding was the systematic direction of discordance. Discordant results were represented by LAMP-positive/contact plate-negative samples, whereas no LAMP-negative/contact plate-positive results were observed. This pattern is relevant from both a statistical and microbiological perspective. Statistically, it explains the significant McNemar tests observed for *S. aureus* with manual extraction, *P. aeruginosa* with manual extraction, and *S. aureus* with automated extraction. Microbiologically, it suggests that LAMP may identify target-specific signals that are not recovered by routine contact plate culture [20]. Such results should therefore be interpreted as discordant findings rather than simply as false-positive molecular results, especially when supported by selective culture verification [21].

### 3.2. Interpretation of Diagnostic Performance and Agreement Metrics

The diagnostic performance profile observed in this study is typical of a rapid molecular assay applied to complex environmental matrices. No LAMP-negative/culture-positive results were observed in the evaluable comparisons; however, PPA estimates were imprecise in subgroups with very few comparator-positive samples. However, several target pathogen subgroups included only a small number of culture-positive samples, particularly *A. baumannii* and *K. pneumoniae*, and the automated extraction subgroups for *K. pneumoniae* and *P. aeruginosa*. Consequently, sensitivity estimates of 100% should be interpreted together with the underlying denominators and confidence intervals. In these low-prevalence subgroups, a single positive sample strongly affects performance estimates, and wider uncertainty is expected.

Specificity (NPA) remained consistently high across targets, but positive predictive value varied according to pathogen prevalence and extraction workflow. This was particularly evident for *K. pneumoniae* and *P. aeruginosa* with automated extraction, where PPV values were low despite high specificity and high overall accuracy. The low PPV values observed in some automated extraction subgroups, particularly for *K. pneumoniae* and *P. aeruginosa*, should be interpreted in the context of the very small number of contact plate-positive observations. Such low positivity is compatible with routinely monitored hospital environments subjected to established cleaning and disinfection procedures and makes PPV estimates highly sensitive to even a few discordant results. Moreover, the two methods differ in their recovery principles. Contact plates provide direct culture on a solid medium from a limited surface area, whereas the LAMP workflow relies on swab sampling followed by 12–16 h enrichment in a liquid medium before molecular detection. Under conditions of low initial target burden, direct solid-medium culture may therefore fail to recover detectable colonies, while a small number of viable organisms collected by swabbing may proliferate during liquid enrichment and subsequently become detectable by LAMP. This interpretation is supported, for microbiologically confirmed samples, by selective culture of the enrichment broth followed by MALDI-TOF MS identification of the corresponding target organism. Accordingly, LAMP-positive/contact plate-negative results should not automatically be classified as molecular false positives. However, because the initial microbial burden was not quantitatively assessed, the contribution of low-level contamination to individual discordant results cannot be established definitively.

Agreement analysis helped to clarify this issue. Cohen’s kappa ranged from moderate to excellent in the manual extraction subgroup and was lower in some automated extraction comparisons, especially when target prevalence was very low. This behavior is expected because Cohen’s kappa is sensitive to prevalence imbalance and may underestimate agreement when most samples are negative. For this reason, PABAK was also reported. PABAK remained high across all comparisons, supporting substantial overall agreement after accounting for the imbalance between positive and negative results. Therefore, Cohen’s kappa and PABAK should be interpreted jointly rather than in isolation.

The McNemar exact test provided additional information by assessing the symmetry of paired discordant results. Significant *p*-values did not demonstrate equivalence or non-equivalence between methods; rather, they indicated that discordance was asymmetric and driven by LAMP-positive/contact plate-negative results. Non-significant McNemar tests, particularly in subgroups with few discordant pairs, should not be interpreted as evidence of equivalence. Overall, the statistical analysis shows that LAMP and contact plate culture are not simply interchangeable methods, but complementary approaches that capture partially different microbiological information.

### 3.3. Manual Versus Automated Extraction Workflow

The stratified analysis by extraction workflow showed high performance for both manual and automated extraction. Manual extraction produced high accuracy, high NPV, and good-to-excellent agreement for all four targets, with Cohen’s kappa values ranging from 0.760 to 0.876 and PABAK values from 0.882 to 0.961. Automated extraction also showed high specificity and NPV, but agreement metrics and PPV were lower for some pathogens because the number of culture-positive samples was very small. The updated *P. aeruginosa* automated extraction data showed one true-positive result, three LAMP-positive/contact plate-negative discordant results, and no false negatives, making sensitivity estimable in this subgroup.

These findings should not be used to infer the superiority of one extraction approach over the other, because the study was not designed as a direct paired comparison of manual and automated extraction on the same individual samples. Rather, the data indicate that both workflows are operationally feasible and can be adapted to different laboratory settings. Automated extraction may improve standardization, reproducibility, and contamination control, whereas manual extraction remains a practical option where automation is not available or where sample volumes are limited. Future studies specifically designed to compare extraction methods in a paired format would be useful to define the most efficient implementation strategy.

### 3.4. Comparison with Previous Literature

The present findings are consistent with previous LAMP studies showing high performance after pre-enrichment. Controlled experiments demonstrated target-dependent effects of incubation time, while later hospital studies reported sensitivity of 1.00 and specificity above 0.90 [18]. The current study adds a larger multicentre sample, including four clinically relevant target species, and directly examines manual and automated extraction workflows. Its novelty therefore lies primarily in pragmatic implementation rather than in first demonstrating the analytical feasibility of LAMP on surfaces.

In the broader literature, LAMP has been applied to the environmental monitoring of water and air, where it has been shown to provide rapid and sensitive detection of microbial indicators and specific pathogens [22,23]. Notably, recent work has used LAMP for *SARS-CoV-2* surface monitoring and comparison with RT-qPCR and droplet digital PCR, highlighting its suitability for point-of-care or on-site environmental testing [24]. Our study contributes novel data on bacterial nosocomial pathogens, demonstrating that LAMP can maintain high performance in complex hospital environments and across multiple institutions.

From an infection-control standpoint, several reviews have emphasized the need for multimodal environmental monitoring, combining visual inspection, ATP bioluminescence, and microbiological methods [8,13,25,26]. The slow turnaround of traditional culture has been identified as a major limitation, potentially delaying feedback on cleaning staff and the implementation of corrective measures. Our data support the integration of LAMP into these multimodal strategies as a rapid microbiological component, capable of providing actionable results during or shortly after cleaning activities.

### 3.5. Implications for Environmental Monitoring and Infection Prevention

The findings have direct implications for hospital hygiene programs and infection prevention practice. Conventional environmental culture requires 48–72 h before colonies can be counted and identified, which delays feedback to cleaning teams and infection-control personnel. In contrast, after the pre-incubation step, the LAMP analytical phase can provide pathogen-specific results within a shortened workflow. This may allow earlier identification of residual contamination, faster reinforcement of cleaning procedures, targeted re-cleaning of high-touch surfaces, and more timely evaluation of sanitation effectiveness.

The clinical value of such a workflow is particularly relevant in very high- and high-risk areas, including intensive care units, neonatal intensive care units, infectious diseases wards, hematology, oncology, surgical areas, and other settings involving caring for vulnerable patients. In these environments, even low-level contamination by pathogens such as *S. aureus*, *A. baumannii*, *K. pneumoniae*, or *P. aeruginosa* may be epidemiologically relevant. Rapid, target-specific environmental screening could therefore function as an early-warning tool within risk-based cleaning and outbreak-prevention strategies.

The detection of LAMP-positive/contact plate-negative results is one of the most important practical findings of this study. These discordant results may reflect bacterial loads below the recovery threshold of contact plates, uneven distribution of contamination on surfaces, reduced culturability after exposure to disinfectants, microbial stress, masking by non-target flora, or molecular detection of residual DNA. The selective verification protocol strengthened interpretation by confirming several LAMP-positive findings on targeted media, especially when routine non-selective culture was negative or difficult to interpret. In practice, this suggests that LAMP could be used as a rapid screening method, with selective culture reserved for positive or epidemiologically relevant samples.

The proposed workflow is therefore best understood as complementary to existing environmental monitoring frameworks rather than as a complete replacement for culture. Culture remains necessary when quantitative CFU data, viable isolates, susceptibility testing, or molecular typing are required. Conversely, LAMP may be most useful when rapid yes/no information is needed to guide immediate infection-control decisions. Integrating both approaches may provide a more complete assessment of environmental risk than either method alone.

### 3.6. Strengths and Limitations

This study has several strengths. It was conducted in three real hospital settings rather than under exclusively experimental conditions, included a relatively large number of surface samples, and focused on clinically relevant pathogens with established roles in healthcare-associated infections. The use of paired LAMP swabs and adjacent RODAC/contact plates enabled direct comparison between molecular and culture-based methods under the same environmental conditions. The stratified performance analysis by pathogen and extraction workflow, together with agreement metrics, PABAK, McNemar testing, and descriptive discordant-result analysis, provides a more complete statistical framework than simple sensitivity and specificity estimate alone.

This study also incorporated a selective verification protocol for LAMP-positive samples. This is an important methodological feature because contact plate culture is an imperfect comparator for environmental surveillance. Contact plates sample a limited area, may be affected by surface geometry and residual disinfectants, and may fail to recover stressed or low-abundance organisms. Selective culture of enrichment broths allowed additional microbiological interpretation of LAMP-positive/contact plate-negative findings and helped distinguish statistical discordance from potentially meaningful biological detection.

Several limitations must also be acknowledged. First, only four bacterial species were included; therefore, the findings cannot be extended to other relevant environmental organisms such as *Enterococcus spp.*, *Clostridioides difficile*, fungi, or respiratory viruses. Second, LAMP provides qualitative results and does not quantify microbial burden in CFUs, which limits its use for threshold-based environmental standards. Third, LAMP does not provide antimicrobial susceptibility data or isolates for epidemiological typing. Fourth, molecular assays may detect DNA from non-viable organisms, especially after cleaning and disinfection. Although the pre-enrichment and selective verification steps reduce this concern, they do not eliminate it.

An additional limitation is the structure of the available dataset. The analysis was based on target-specific aggregated comparisons rather than a complete individual-level dataset including all clustering variables for each sample-target observation. For this reason, formal mixed-effects modelling was not performed, and centre-level patterns were interpreted descriptively. In addition, sanitation-related variables were not available for sample-level analysis; therefore, the potential effect of cleaning and disinfection procedures on LAMP or culture positivity could not be assessed. The present study was not designed to evaluate the efficacy of specific sanitation procedures, and the findings should therefore not be interpreted as evidence of the effectiveness of individual cleaning or disinfection protocols. Future studies using individual-level data and standardized sanitation-related metadata will be needed to investigate these effects.

### 3.7. Future Directions

Future research should focus on prospective implementation studies designed to assess whether LAMP-based environmental surveillance improves infection-prevention outcomes. Longitudinal studies could evaluate whether rapid LAMP feedback reduces response time after detection of contamination, improves compliance with cleaning protocols, and contributes to reducing environmental reservoirs during outbreaks. Economic evaluations are also warranted to compare LAMP-based workflows with conventional culture, ATP monitoring, and combined multimodal surveillance strategies.

Further technological development should include broader LAMP panels targeting additional nosocomial pathogens and resistance markers, such as carbapenemase genes or methicillin-resistance determinants. Semi-quantitative LAMP outputs, time-to-positivity thresholds, or combined LAMP–culture algorithms may help define actionable contamination thresholds. Finally, integration of LAMP results into digital dashboards could support real-time visualization of environmental risk, traceability of corrective actions, and feedback to frontline staff. In this perspective, LAMP should be viewed not only as a diagnostic test, but as a rapid operational tool within modern infection prevention and environmental hygiene programs.

## 4. Materials and Methods

### 4.1. Study Design and Setting

This multicentre observational method-comparison study was designed to evaluate the diagnostic performance and operational feasibility of loop-mediated isothermal amplification (LAMP) for the rapid detection of selected nosocomial pathogens on hospital environmental surfaces. Environmental samples were analysed in parallel using the Enbiotech LAMP system and a conventional culture-based (contact plate) method, which was used as the microbiological comparator for the primary diagnostic–performance analysis. Sampling sites were selected according to local environmental monitoring plans and risk-based criteria derived from national hospital sanitation guidance and UNI EN 17141:2021 (UNI EN 17141:2021; Cleanrooms and Associated Controlled Environments—Biocontamination Control. UNI—Ente Italiano di Normazione: Milan, Italy, 2021.) for the microbiological control of controlled environments. The sampling campaign was conducted from January 2024 to December 2025.

**Figure 1:**
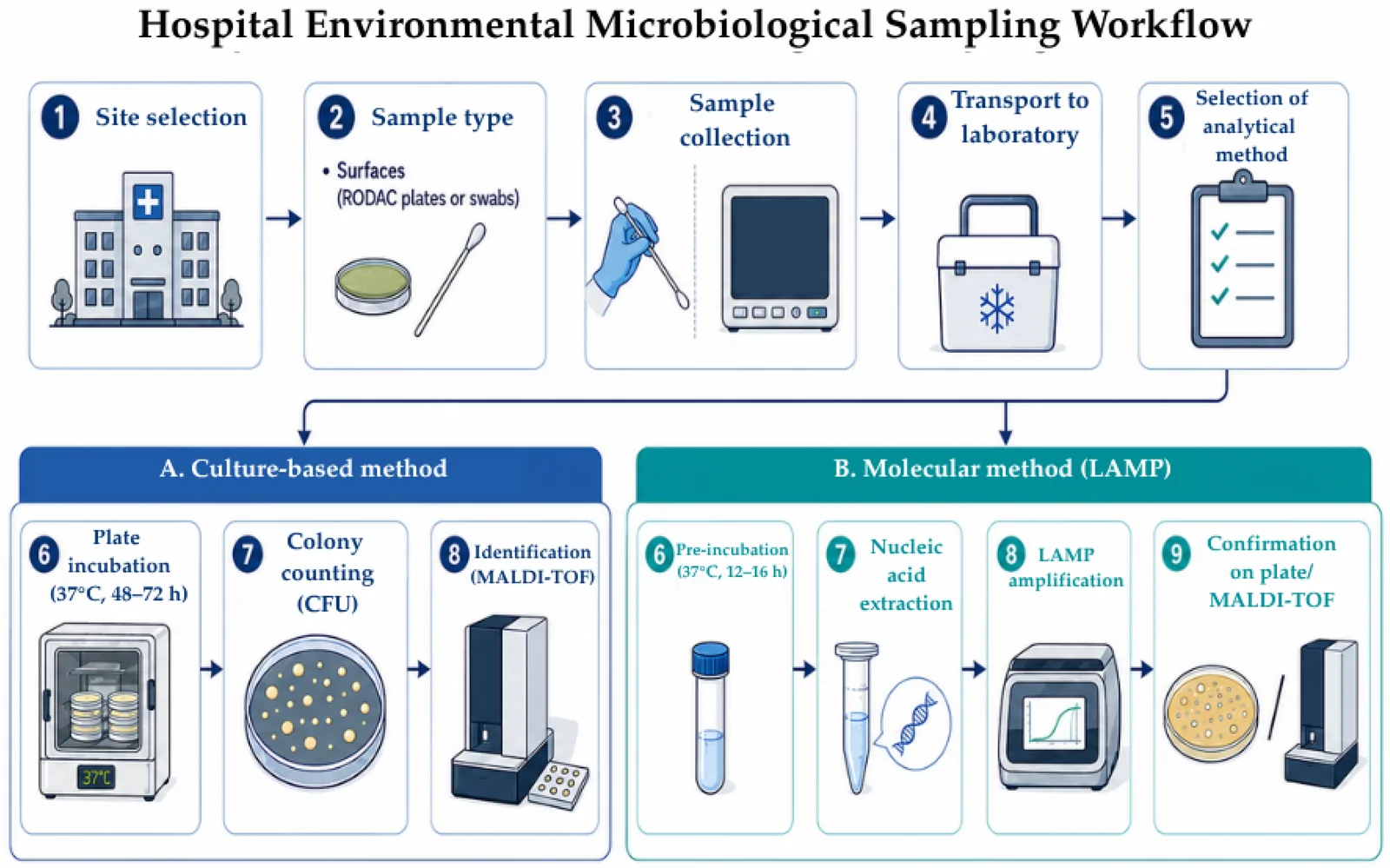
Hospital environmental microbiological sampling workflow.

Three Italian hospital and laboratory centres participated in the study, representing different geographical and organizational settings: ARNAS Garibaldi of Catania (Sicily centre; 497-bed hospital; microbiological analyses performed at the Department of Biomedical and Biotechnological Sciences), “A.O.U. San Giovanni di Dio e Ruggi d’Aragona”, Salerno (Campania centre; 642-bed teaching hospital; environmental sampling and processing coordinated by the Hospital Hygiene and Epidemiology Unit), and AUSL Romagna—“DiMeLabeT” (Emilia-Romagna centre; 3350-bed hospital; laboratory work conducted at the Department of Laboratory Medicine and Transfusion). All centres followed the same procedure of sampling and laboratory protocol shared within the network, according to the manufacturer’s guidelines (Figure 1).

### 4.2. Target Pathogens and LAMP Assays

This study focused on four bacterial species selected for their clinical relevance in healthcare-associated infections, environmental persistence, and potential involvement in hospital outbreaks: *Staphylococcus aureus*, *Acinetobacter baumannii*, *Klebsiella pneumoniae*, and *Pseudomonas aeruginosa*. Pathogen-specific commercial LAMP kits supplied by Enbiotech s.r.l. (Palermo, Italy) were used according to the manufacturer’s instructions. Each assay provided a qualitative positive/negative result for the corresponding target pathogen. Positive and negative controls supplied with the kits were included in each analytical session and only runs with valid controls were considered interpretable.

### 4.3. Environmental Surface Sampling

A total of 298 environmental surface samples were collected during the study period, including 132 samples from Campania, 93 from Emilia-Romagna, and 73 from Sicily. Sampling was performed according to the routine infection-prevention and environmental microbiological monitoring procedures adopted by the participating centres. Sanitation-related variables were not included in the present method-comparison analysis because cleaning and disinfection procedures, including their timing and operational implementation, were determined by local hospital protocols and were not standardized for the purposes of this study. High-touch and near-patient surfaces were prioritized when defined by the local monitoring plan, because these surfaces are most relevant for environmental transmission risk and for evaluating sanitation procedures.

For each sampling point, a paired environmental sampling strategy was applied. For the molecular workflow, a flocked swab moistened with neutralizing solution was rubbed over a 10 cm × 10 cm area of the selected surface using a standardized multidirectional pattern. The swab was then placed into a tube containing enrichment medium. A second dry flocked swab was passed over the same area and added to the same tube to increase recovery from the sampled surface. Tubes were vortexed, labelled, transported to the laboratory under controlled conditions, and incubated at 37 °C for 12–16 h before nucleic acid extraction and LAMP analysis.

For the culture-based comparator, a RODAC/contact plate containing TSA-LTH medium was applied to a 25 cm^2^ area adjacent to the LAMP sampling site using uniform pressure for 10 s. Contact plates were incubated at 37 °C for 48–72 h. After incubation, colonies were counted and target colonies were identified to species level by MALDI-TOF mass spectrometry. The use of adjacent swab and contact plate sampling allowed a paired comparison under the same environmental conditions, while minimizing interference between the two sampling procedures.

### 4.4. Culture-Based Comparator and Definition of Primary Diagnostic Outcomes

The primary microbiological comparator consisted of conventional RODAC/contact plate culture followed by species identification. For the primary diagnostic-performance analysis, each pathogen-specific comparison was classified using a 2 × 2 contingency table. A true positive was defined as a LAMP-positive result in a sample that was also positive for the same target pathogen by contact plate culture. A false positive was defined, for the primary comparison, as a LAMP-positive/contact plate-negative result. A true negative was defined as concordant negativity by both methods, whereas a false negative was defined as a LAMP-negative/contact plate-positive result. Because LAMP provides qualitative results, the comparison was based on the presence or absence of each target pathogen rather than on quantitative CFU thresholds.

Contact plate culture also provided semi-quantitative information on surface microbial burden and allowed recovery of viable isolates for identification. Therefore, culture was retained as the reference comparator for conventional environmental monitoring, even though it was not considered an absolute gold standard for all biologically plausible contamination events. This distinction was important for interpreting LAMP-positive/contact plate-negative discordant results.

### 4.5. LAMP Pre-Enrichment, Nucleic Acid Extraction, and Amplification

Following overnight pre-enrichment, nucleic acids were extracted using either a manual or an automated workflow according to the Enbiotech ICA Panel protocol.

For manual extraction, 1.6 mL of the pre-enriched sample was transferred into a 2 mL secure-lock tube and centrifuged at 16,000× *g* for 5 min. The supernatant was discarded without disturbing the pellet, which was resuspended in 200 µL of 1× TE buffer supplied with the Enbiotech kit (Enbiotech srl, Palermo, Italy). Samples were then incubated at 95 ± 1 °C for 10 min for thermal lysis, followed by centrifugation at a maximum speed for 20 s. The resulting nucleic acid extract was directly used for LAMP amplification.

For automated extraction, the BIOCOMMA^®^ automated extraction platform was used with the Enbiotech magnetic bead extraction kit (Cat. No. EBT 110A 1303/SCL, Enbiotech srl, Palermo, Italy). After vortexing the magnetic beads, 40 µL were added to the designated wells of the extraction plate, together with 800 µL of pre-enriched sample. The automated ICA_O.N. extraction program was then performed according to the manufacturer’s protocol, using the corresponding washing and elution steps. At the end of the extraction cycle, the eluate was recovered from the designated elution wells and used directly for LAMP analysis.

For amplification, 14 µL of LAMP Mix was added to each pathogen-specific Primer Mix tube, followed by 6 µL of extracted nucleic acid. Reactions were performed on the ICGENE^®^ platform (Enbiotech srl, Palermo, Italy) under the manufacturer-recommended isothermal conditions. Amplification was monitored in real time, and results were automatically classified as positive, negative, or invalid (“ERROR”) according to the target-specific fluorescence signal and the internal control. Positive results indicated the presence of the corresponding target nucleic acid, whereas negative results required successful amplification of internal control.

Because allocation to manual or automated extraction reflected the operational organization of the participating centres, the two workflows were analysed descriptively and were not interpreted as fully paired analytical conditions.

### 4.6. Selective Verification of LAMP-Positive Findings

To support the microbiological interpretation of discordant results, a selective plating verification protocol was applied to enrichment broths corresponding to LAMP-positive samples, particularly when the paired contact plate did not recover the same target pathogen. Aliquots from the enrichment broths were plated onto target-selective media: Baird-Parker agar for *Staphylococcus aureus*, Acinetobacter selective medium (LAM) for *Acinetobacter baumannii*, MacConkey agar for *Klebsiella pneumoniae*, and P.A.CN selective agar for *Pseudomonas aeruginosa*. Plates were incubated at 37 °C for 24–48 h, and suspected target colonies were identified by MALDI-TOF mass spectrometry when required.

Selective verification was used as a secondary microbiological support tool for discordant-result interpretation. It was not used to replace the primary RODAC/contact plate comparator in the diagnostic-performance table, but it provided additional evidence that some LAMP-positive/contact plate-negative results represented target organisms recoverable after enrichment and selective culture.

### 4.7. Statistical Analysis

All statistical analyses were performed using R software (version 4.5.3) (R Foundation for Statistical Computing, Vienna, Austria). Qualitative LAMP results obtained with the Enbiotech system were compared with conventional RODAC/contact plate culture, which was used as the microbiological comparator method. For each target pathogen and extraction method, results were arranged in 2 × 2 contingency tables and classified as true positives (TPs), false positives (FPs), true negatives (TNs), and false negatives (FNs). Analytical performance was assessed by calculating relative sensitivity/positive percent agreement (PPA), relative specificity/negative percent agreement (NPA), overall accuracy/overall percent agreement (OPA), positive predictive value (PPV), negative predictive value (NPV), F1-score, and balanced accuracy. Exact 95% confidence intervals for binomial proportions were calculated using the Clopper–Pearson method when appropriate.

Agreement between LAMP and contact plate culture was evaluated using Cohen’s κ coefficient. Because several pathogen-specific subgroups had a low prevalence of positive samples and highly unbalanced positive/negative distributions, prevalence-adjusted bias-adjusted kappa was also calculated. The exact McNemar test was used to assess whether discordant paired results were symmetrically distributed between LAMP-positive/contact plate-negative and LAMP-negative/contact plate-positive outcomes. A two-sided *p*-value < 0.05 was considered statistically significant. McNemar results were interpreted as evidence of asymmetric discordance and not as evidence of analytic equivalence or non-equivalence between methods.

The absolute difference in positivity rates between LAMP and contact plate culture was also calculated for each pathogen and extraction workflow. Discordant results were analysed descriptively, with particular attention to LAMP-positive/contact plate-negative samples and to the results of selective verification. Because the available dataset was based on aggregated target-specific contingency counts rather than complete individual-level observations including all clustering variables, formal mixed-effects modelling was not performed. Centre-level and ward-level patterns were therefore interpreted as exploratory descriptive analyses.

## 5. Conclusions

This multicentre study evaluated the agreement and operational feasibility of a LAMP-based workflow for the detection of four clinically relevant nosocomial pathogens on hospital environmental surfaces under real-world conditions. LAMP showed high agreement with the culture-based comparator, and no LAMP-negative/culture-positive results were observed in the evaluable comparisons. However, the small number of comparator-positive samples in some pathogen-specific subgroups limits the precision of PPA estimates and should be considered when interpreting these findings.

The LAMP workflow provided pathogen-specific qualitative results within a shorter analytical timeframe than conventional culture, although following a 12–16 h pre-enrichment step. These findings support the potential use of LAMP as a complementary tool for hospital environmental surveillance. Culture remains necessary when quantitative microbial burden, viable isolates, antimicrobial susceptibility testing, or further strain characterization are required. Further prospective studies are needed to determine whether implementation of this approach translates into measurable improvements in environmental hygiene or infection-prevention outcomes. At the same time, LAMP should not be viewed as a complete replacement for culture-based monitoring. Conventional culture remains indispensable for CFU quantification, the recovery of viable isolates, antimicrobial susceptibility testing, and epidemiological typing. The most appropriate implementation is therefore a complementary workflow in which LAMP provides rapid target-specific screening, while culture confirms positive or epidemiologically relevant results and provides isolate-based characterization.

Future prospective studies using individual-level data should assess the impact of LAMP-based environmental monitoring on cleaning performance, outbreak preparedness, turnaround time, cost-effectiveness, and HAI-related outcomes. Expansion of target panels to include additional pathogens and resistance markers could further strengthen their role in integrated infection prevention and environmental hygiene programmes.

## Figures and Tables

**Table 1:** Paired agreement between LAMP and contact plate culture according to target pathogen and nucleic acid extraction workflow.

Extraction	Target	Valid Test	LAMP+ /Culture+	LAMP+ /Culture−	LAMP−/Culture+	LAMP− /Culture−	PPA (n/N; 95% CI)	NPA (n/N; 95% CI)
Manual	*S. aureus*	153	54	9	0	90	100% (54/54; 93.4–100)	90.9% (90/99; 83.4–95.8)
Manual	*A. baumannii*	153	5	3	0	145	100% (5/5; 47.8–100)	98.0% (145/148; 94.2–99.6)
Manual	*K. pneumoniae*	153	7	4	0	142	100% (7/7; 59.0–100)	97.3% (142/146; 93.1–99.2)
Manual	*P. aeruginosa*	153	20	7	0	126	100% (20/20; 83.2–100)	94.7% (126/133; 89.5–97.9)
Automated	*S. aureus*	145	6	9	0	130	100% (6/6; 54.1–100)	93.5% (130/139; 88.1–97.0)
Automated	*A. baumannii*	145	2	2	0	141	100% (2/2; 15.8–100)	98.6% (141/143; 95.0–99.8)
Automated	*K. pneumoniae*	145	1	4	0	140	100% (1/1; 2.5–100)	97.2% (140/144; 93.0–99.2)
Automated	*P. aeruginosa*	145	1	3	0	141	100% (1/1; 2.5–100)	97.9% (141/144; 94.0–99.6)

PPA, positive percent agreement; NPA, negative percent agreement; and CI, confidence interval. Contact plate culture was used as the microbiological comparator rather than an absolute gold standard. Exact 95% CIs were calculated using the Clopper–Pearson method.

## Data Availability

All data were reported in this version of manuscript.

## Supplementary Materials

The following supporting information can be downloaded at: https://www.mdpi.com/article/10.3390/antibiotics15101012/s1, Table S1: Secondary performance and agreement metrics for LAMP versus contact-plate culture according to target pathogen and extraction workflow; Table S2: Distribution of samples and valid paired comparisons according to participating centre, extraction workflow, and target pathogen.

## Abbreviations

CFUs	Colony-forming units
DNA	Deoxyribonucleic acid
FNs	False negatives
FPs	False positives
HAIs	Healthcare-associated infections
IPC	Infection prevention and control
LAMP	Loop-mediated isothermal amplification
MALDI-TOF MS	Matrix-assisted laser desorption/ionization time-of-flight mass spectrometry
NPA	Negative percent agreement
NPV	Negative predictive value
OPA	Overall percent agreement
PABAK	Prevalence-adjusted bias-adjusted kappa
PCR	Polymerase chain reaction
PPA	Positive percent agreement
PPV	Positive predictive value
RODAC	Replicate organism detection and counting
TAT	Turnaround time
TNs	True negatives
TPs	True positives
TSA-LTH	Tryptic soy agar with lecithin, Tween 80 and histidine
WHO	World Health Organization
κ	Cohen’s kappa coefficient
