# Supplementary material for: Multicentre Evaluation of Loop-Mediated Isothermal Amplification (LAMP) for Environmental Monitoring of Nosocomial Pathogens in Italian Hospitals
Source: Antibiotics (Basel). 2026 Oct 12;15(10):1012. doi: 10.3390/antibiotics15101012 (PMC13700765; doi:10.3390/antibiotics15101012)
Supplement: Supplementary file 1 [file antibiotics-15-01012-s001.zip › antibiotics-4508800-supplementary.pdf]

Supplementary Table S1. Secondary performance and agreement metrics for LAMP versus contact-plate culture according to target pathogen and extraction workflow.

Extraction workflow	Target pathogen	Valid pairs, n	OPA	PPV	NPV	F1-score	Balanced accuracy	Cohen's κ	PABAK	Exact McNemar p
Manual	*S. aureus*	153	94.1%	85.7%	100.0%	92.3%	95.5%	0.876	0.882	0.004
Manual	*A. baumannii*	153	98.0%	62.5%	100.0%	76.9%	99.0%	0.760	0.961	0.250
Manual	*K. pneumoniae*	153	97.4%	63.6%	100.0%	77.8%	98.6%	0.765	0.948	0.125
Manual	*P. aeruginosa*	153	95.4%	74.1%	100.0%	85.1%	97.4%	0.825	0.908	0.016
Automated	*S. aureus*	145	93.8%	40.0%	100.0%	57.1%	96.8%	0.545	0.876	0.004
Automated	*A. baumannii*	145	98.6%	50.0%	100.0%	66.7%	99.3%	0.660	0.972	0.500
Automated	*K. pneumoniae*	145	97.2%	20.0%	100.0%	33.3%	98.6%	0.326	0.945	0.125
Automated	*P. aeruginosa*	145	97.9%	25.0%	100.0%	40.0%	99.0%	0.393	0.959	0.250

OPA, overall percent agreement; PPV, positive predictive value; NPV, negative predictive value; PABAK, prevalence-adjusted bias-adjusted kappa. PPV and NPV are comparator-relative descriptive measures. PABAK is reported as a secondary agreement measure and should be interpreted cautiously because the predominance of negative observations may contribute to high agreement estimates. The exact McNemar test evaluates directional asymmetry among discordant paired results; $p < 0.05$ was considered statistically significant.

Supplementary Table S2. Distribution of samples and valid paired comparisons according to participating centre, extraction workflow, and target pathogen.

Centre	Region	Extraction workflow	Samples allocated to workflow, n	*S. aureus*	*A. baumannii*	*K. pneumoniae*	*P. aeruginosa*
Cesena	Emilia-Romagna	Manual	47	47	47	47	47
Catania	Sicily	Manual	32	32	32	32	32
Salerno	Campania	Manual	74	74	74	74	74
**Manual subtotal**	—	**Manual**	153	153	153	153	153
Cesena	Emilia-Romagna	Automated	46	46	46	46	46
Catania	Sicily	Automated	41	41	41	41	41
Salerno	Campania	Automated	58	58	58	58	58
Automated subtotal	—	Automated	145	145	145	145	145
Overall	—	—	298	298	298	298	298

Values indicate the number of environmental samples allocated to each extraction workflow and the corresponding valid paired LAMP/contact-plate comparisons for each target pathogen. In the corrected dataset, all 153 manual-extraction samples and all 145 automated-extraction samples yielded valid paired comparisons for each of the four targets (overall n = 298). Workflow allocation reflected the operational organization of the participating centres and did not constitute a fully paired or randomized comparison of extraction methods. Complete harmonized individual-level distributions by ward, surface type, sanitation status, and sampling period were not available for all observations.
